# Die Kataraktchirurgie und das kleine Auge: Relativer anteriorer Mikrophthalmus, hohe Hyperopie und Nanophthalmus

**DOI:** 10.1007/s00347-021-01483-5

**Published:** 2021-08-27

**Authors:** Gerd U. Auffarth, Tadas Naujokaitis, Maximilian Hammer

**Affiliations:** Augenklinik der Universität Heidelberg, Im Neuenheimer Feld 400, 69120 Heidelberg, Deutschland

**Keywords:** Komplizierte Katarakt-Chirurgie, IOL-Berechnung, Einfacher Mikrophthalmos, Phakoemulsifikation, Vorderabschnitt Anatomie, Ko-Morbiditäten, Complex cataract surgery, IOL calculation, Simple microphthalmos, Phacoemulsification, Anterior segment anatomy, Comorbidities

## Abstract

Relativer anteriorer Mikrophthalmus, hochgradige Hyperopie und Nanophthalmus bezeichnen klein gebaute Augen mit unterschiedlichem morphologischem Verhältnis zwischen Vorderabschnitt und Achsenlänge. Im Rahmen dieses Beitrags werden intraoperative Herausforderungen und chirurgische Lösungsansätze für die Kataraktoperation bei Patienten mit einer der 3 genannten morphologischen Veränderungen diskutiert. Zusätzlich wird auf mögliche, vorliegende Komorbiditäten, wie z. B. das Glaukom, und die präoperative Planung eingegangen.

**Video online:**

Die Online-Version dieses Beitrags (10.1007/s00347-021-01483-5) enthält ein Video.

## Abstract (en)

Relative anterior microphthalmos, nanophthalmos and high-grade hyperopia are small eyes with different characteristic morphological relationships between the anterior segment and axis length. This article discusses the intraoperative challenges and surgical approaches to solutions for cataract operations in patients with one of the three named morphological alterations. Additionally, the article addresses possible comorbidities including glaucoma and preoperative planning.

Im klinischen Alltag begegnen uns nicht selten „klein gebaute“ Augen: Der relative anteriore Mikrophthalmus (RAM), die hochgradige Hyperopie und der Nanophthalmus. Außer der Größenverhältnisse sind die Augen morphologisch intakt und voll funktionsfähig [10]. Diese 3 Krankheitsbilder werden oft unter dem Begriff „einfacher Mikrophthalmus“ zusammengefasst und sind abzugrenzen von genetischen Missbildungen mit funktionellen Störungen, die mit einem Mikrophthalmus einhergehen (komplexer Mikrophthalmus). Dieser Beitrag behandelt Techniken und Lösungsansätze für die Kataraktchirurgie bei Patienten mit einfachem Mikrophthalmus. Wird die Morphologie präoperativ erkannt, können Techniken wie die Soft-Shell-Technik, Posterior-Plane-Emulsifikation, Step-by-Step-Chop, aber auch die angepasste Berechnung der Brechkraft der Intraokularlinse (IOL) zum Einsatz kommen, um bestmögliche Ergebnisse zu erzielen. Die Tab. 1 gibt eine Übersicht über die Inhalte des Artikels.

**Table 1:** 

Herausforderung	Lösungsansatz
*IOL-Berechnung*	
Ungenaue IOL-Berechnung	Patient informieren und Erwartungen anpassen
Hoher postoperativer refraktiver Fehler wahrscheinlich	Korrektion des refraktiven Fehlers postoperativ
Wenige Studien für IOL-Berechnung in extrem kurzen Augen	Vorsicht bei der Übertragung von Studien in kurzen Augen auf extrem kurze Augen
Hohe Diskrepanz zwischen Formeln	Vergleich der Ergebnisse mehrerer Formeln
*High-power-Intraokularlinsen*	
Toleranz von ± 1,0 dpt für das IOL-Power-Labeling	Diese Quelle eines postoperativen refraktiven Fehlers im Kopf behalten
Erhöhte sphärische Aberration	Verwendung von asphärischen IOLs
*Intra- und postoperative Überlegungen*	
Möglicher lentikulärer Block/Glaukomanfall	Präoperative Drucksenkung mit Osmofundin und Acetazolam (i.v.)
	Postoperative Kontrolle des intraokularen Drucks
Suboptimaler Zugang	Temporaler Zugang
Hohe Komplikationsrate	Adäquate Vorbereitung und Informieren des Patienten
Erhöhtes Risiko für uveale Effusion	Vollnarkose
Hornhautödem	Soft-Shell-Technik
Hinterer Kapselriss	Posterior-Plane-Emulsifikation

## Definitionen

**Figure 1:**
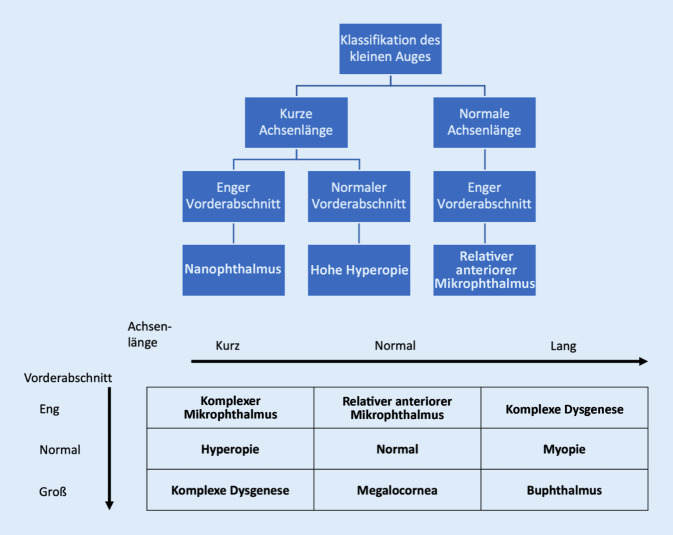

Zur Unterscheidung der Unterformen des einfachen Mikrophthalmus werden 2 Größen herangezogen: die Achsenlänge und die Größe des Vorderabschnitts. Die Abb. 1 zeigt diese Zusammenhänge auf. Während beim RAM die Achsenlänge nicht auffällig ist (> 20 mm), ist der Vorderabschnitt disproportional klein, definiert durch einen horizontalen Durchmesser der Hornhaut < 11 mm und einer Tiefe des Vorderabschnitts von rund 2 mm. Liegt ein normaler Vorderabschnitt vor, aber eine verkürzte Achsenlänge, so liegt eine Hyperopie vor. Der Nanophthalmus ist durch ein in der Gesamtheit verkleinertes Auge mit einer Achsenlänge < 20 mm oder < 20,5 mm definiert [2].

## Relativer anteriorer Mikrophthalmus

RAM ist eine häufig nicht erkannte anatomische Besonderheit. Bei ungefähr 6 % der Bevölkerung liegt ein RAM vor, RAM ist damit deutlich häufiger als der Nanophthalmus oder die hochgradige Hyperopie. Bei einem präoperativen Screening müssen Vorderabschnitt und Achsenlänge vermessen werden, um Komplikationen vorzubeugen. Ist ein RAM diagnostiziert, ermöglicht die chirurgische Planung, viele der Probleme zu umgehen und vorbereitet zu sein.

Bei RAM liegt die summierte Prävalenz der Glaukomformen bei 77 %. Ein Großteil der Patienten wurde zum Zeitpunkt der Kataraktoperation bereits mit invasiver Glaukomtherapie versorgt. (62 %) Neben den Glaukomen liegen oft Cornea guttata (45 %) und überdurchschnittlich oft hintere Synechien sowie ein Pseudoexfoliationssyndrom vor (12 %) [2, 10].

## Hochgradige Hyperopie

Bei der hochgradigen Hyperopie liegen meist ein morphologisch normaler Vorderabschnitt und ein verkürzter Hinterabschnitt vor. Die Hyperopie kann in 3 Schweregrade, basierend auf dem refraktiven Fehler, eingeteilt werden: geringgradige Hyperopie mit maximal +2 dpt, mittelgradige Hyperopie mit +2,25 dpt bis +5 dpt und hochgradige Hyperopie mit über +5 dpt. Die Schwierigkeit der IOL-Berechnung steht somit im Vordergrund. Falls der Hinterabschnitt sehr kurz ist, steigt das Risiko des uvealen Effusionssyndroms nach Kataraktchirurgie, eine intraoperative Ablatio ist jedoch sehr selten. Der Übergang zum Nanophthalmus ist fließend.

## Nanophthalmus

Nanophthalmus weist eine sehr geringe Prävalenz mit 0,0009–0,017 % auf. *NNO1* auf Chromosom 11 und *MFRP* auf 11q23.3 sind Gene, die mit Nanophthalmus assoziiert sind. Eine veränderte Kollagenfaseranordnung in diesen Augen führt zu einem erschwerten Wachstum des Auges und einer verdickten und gleichzeitig schwächeren posterioren Sklera, was mit einer Behinderung der venösen Drainage der Vortexvenen mit Risiko einer uvealen Effusion einhergeht. Zusätzlich entsteht durch die normal ausgebildete Linse eine vermehrte Einengung der Vorderkammer, was zum lentikulären Block prädestiniert. Des Weiteren besteht eine erhöhte Wahrscheinlichkeit für intra- und postoperative Komplikationen bei der Kataraktchirurgie in Augen mit kurzer Achsenlänge, wie ein zystisches Makulaödem, choroidale Hämorrhagie, Glaskörperblutungen, Netzhautablösungen, eine Hornhautdekompensation und Kammerwasserabflussstörungen. Unerwünschte Ergebnisse resultieren auf dem Boden der engen Vorderkammermorphologie, der Komorbiditätslast und durch eine erschwerte Berechnung der Zielrefraktion aufgrund der morphologischen Besonderheiten, auf welche die meisten IOL-Formeln nicht kalibriert sind. Zudem liegt das Auge meist tiefer in der Orbita, was den operativen Zugang erschwert.

Der enge Vorderabschnitt ist dem RAM und dem Nanophthalmus gemein. Im Folgenden werden chirurgische Lösungsansätze hierfür besprochen.

## Endothelialer Zellverlust und postoperatives Kornealödem

Probleme, basierend auf der Enge des Vorderabschnitts, können eingeteilt werden in Komplikationen durch eine enge Morphologie/kleinen Hornhautdurchmesser sowie in Komplikationen durch die kleine Pupillengröße.

In vorherigen Studien trat bei Patienten mit RAM, die bereits Cornea guttata oder eine niedrige Endothelzelldichte zeigten, häufiger ein postoperatives Hornhautödem auf als bei einer Kontrollgruppe mit regelrechtem Vorderabschnitt. Höchstwahrscheinlich wird dies durch die Enge des Vorderabschnitts ausgelöst. Im Durchschnitt verloren die Patienten zwischen 11 und 13 % der Endothelzelldichte. Techniken wie die Soft-Shell-Technik nach Arshinoff [1] ermöglichen einen verbesserten Schutz des Endothels. Dispersive und kohäsive Viskoelastika werden in der Vorderkammer gleichzeitig genutzt: Zuerst wird dispersive, im Anschluss kohäsive Substanz in den Vorderabschnitt gegeben. Es bildet sich ein protektiver Film aus dispersivem Viskoelastikum auf den Endothelzellen aus, während der kohäsive Part die Stabilität (raumfüllender Effekt) in der Vorderkammer gewährleistet. Die Technik bietet generell Vorteile bei engem Vorderabschnitt. Insbesondere die Verwendung von hochkohäsiven OVD (z. B. Healon5, Johnson & Johnson, New Brunswick, NJ, USA) sind von großer Bedeutung bei einer flachen Vorderkammermorphologie. Durch die hohe Viskosität und Elastizität [3] können eine bessere Stabilität und Tiefe der Vorderkammer, eine Pupillenerweiterung und eine Abflachung der Linsenvorderfläche während der Kataraktoperation erzielt werden. In extremen Fällen mit sehr engem Vorderabschnitt empfiehlt es sich, eine vordere Vitrektomie zu Beginn der Operation durchzuführen. Die dadurch verbesserte Situation ermöglicht einen komplikationsärmeren Verlauf.

Durch den engen Vorderabschnitt besteht ebenso ein erhöhtes Risiko für einen Einriss der hinteren Kapsel: Meist versucht der Chirurg instinktiv, das Endothel zu schützen, und aspiriert Teile der Linse weiter posterior als gewöhnlich. Vasavada et al. liefern eine mögliche Lösung: die Posterior-Plane-Emulsifikation mit Sculpting [13]. Während des Vorgangs werden gleichzeitig Stärke des Vakuums, Energie und Fluss der Aspiration verringert, um effektiv Schäden der hinteren Kapsel zu vermeiden. Eine Hinterkapselruptur ist in dieser Gruppe an Patienten strengstens zu vermeiden. Die meist individuell hergestellten IOLs liegen in der Regel als einstückige IOLs zur geplanten Kapselsackimplantation vor. Sollte eine Sulkusimplantation nötig werden, kann diese nicht mit der einstückigen IOL durchgeführt werden. Bis zur endgültigen Fixation einer neu gefertigten High-Power-IOL finden Standby-Strategien Anwendung.

## Enger Pupillendurchmesser und Pseudoexfoliation

Die hohe Prävalenz einer kleinen Pupille (< 4 mm) in Kombination mit hinteren Synechien und Pseudoexfoliationssyndrom in dieser Patientenpopulation macht eine mechanische Dilatation der Pupille oft unabwendbar.

Reicht die pharmakologische Dilatation der Pupille während der Operation nicht aus, gibt es mehrere Möglichkeiten zur mechanischen Dilatation: Sphinkterotomie, Irisretraktoren und Pupillenexpander (i-Ring, Malyugin-Ring). Studien zeigten, dass bei richtiger Verwendung eines Irisexpanders hierbei sogar Komplikationen wie Endothelzellverlust verringert werden können.

Trotz mechanischer Dilatation werden in diesen Augen selten optimale Bedingungen geschaffen. Bei der Phakoemulsifikation kann z. B. die Step-by-Step-Chop-Technik nach Vasavada verwendet werden. Die geringstmögliche Manipulation der Iris ist wichtig, da schon vor der Kataraktoperation besonders bei Patienten mit RAM eine ausgeprägte Irisinstabilität vorliegen kann.

## Komorbiditätslast: Implikationen für die Kataraktchirurgie

### Glaukome

Vom Engwinkel- bis zum Offenwinkelglaukom (z. B. sekundäres Pseudoexfoliationsglaukom) können verschiedene Formen des Glaukoms vorliegen. In vielen Fällen mit engem Vorderabschnitt wurde bereits ein Glaukom operativ therapiert. In diesen Fällen ist ein temporaler Zugangsweg oft vorteilhaft. Studien zeigten, dass hierdurch möglicherweise auch ein geringerer Verlust an Hornhautendothel möglich ist. In bestimmten Formen des Nanophthalmus kann ein Glaukomanfall als lentikulärer Block durch die natürliche Linse selbst (und später durch die Kunstlinse) ausgelöst werden. Die Kataraktoperation kann hier als „kausale“ Therapie des Glaukoms erwogen werden, bevor invasive Glaukomverfahren Anwendung finden (antiglaukomatöse Linsenextraktion).

## IOL-Berechnung

Das gute postoperative, refraktive Ergebnis ist in kurz gebauten Augen eine Herausforderung. Seit 1993 wird die Hoffer-Q-Formel als Ausgangspunkt für die IOL-Berechnung in kurzen Augen angesehen [11]. Eine Studie von 2016 in 29 Augen mit RAM zeigte eine postoperative Zielrefraktion ± 1 dpt in 72,4 % mit der Hoffer-Q-Formel. Dies war nur in 46,6 % der Fälle bei den 15 eingeschlossenen, nanophthalmischen Augen der Fall [7].

Eine Reihe an aktuellen Studien konnte keine signifikanten Unterschiede zwischen Hoffer Q, Barrett Universal II, Haigis, Holladay 2 und RBF 1.0 bei der IOL-Berechnung in kurzen Augen zeigen [12]. Es wird empfohlen, bei Patienten mit flacher Vorderkammertiefe (z. B. < 2,4 mm) dies zu beachten. Je flacher die Vorderkammer, desto ungenauer war die IOL-Berechnung basierend auf Hoffer-Q- und Haigis-Formel [4].

Eine neuere Formel, die ebenfalls vielversprechende Ergebnisse in kurzen Augen, insbesondere bei hochgradiger Hyperopie, erzielen konnte, ist die Kane-Formel [8]. Hipólito-Fernandes et al. zeigten, dass unter anderem die Kane-Formel in geringem Maße in ihrem postoperativen Ergebnis durch die Linsendicke und Vorderkammertiefe beeinflusst wird. Im Gegensatz dazu neigte die z. B. Haigis-Formel zu refraktiven Abweichungen bei variierenden Linsendicken [5]. Die Kane-Formel sollte jedoch nur in Kombination mit etablierten Formeln zum Einsatz kommen. Sie wurde erst im September 2017 entwickelt und ist nicht offengelegt, was die externe Validierung erschwert.

Insbesondere bei Patienten mit Nanophthalmus sollten die Ergebnisse mehrerer Formeln verglichen werden. Hierbei können Ausreißer identifiziert werden, die bei alleiniger Verwendung einer Formel zu großen refraktiven Fehlern führen können. Für RAM, Nanophthalmus und hochgradige Hyperopie empfiehlt sich die Verwendung von High-Power-IOLs, die zum Teil individuell hergestellt werden. Die Verwendung von Piggyback-IOL-Implantaten ist in Deutschland obsolet und sollte nur in Regionen angewendet werden, in denen High-Power-IOLs nicht verfügbar sind. Eine der möglichen Fehlerquellen der postoperativen Refraktion basiert jedoch auch auf den High-Power-IOLs: Die zugehörige ISO-Norm erlaubt eine höhere Toleranz der Brechkraft bei Linsen mit hohen Brechwerten und sollte als potenzieller Fehler beachtet werden.

**Figure 2:**
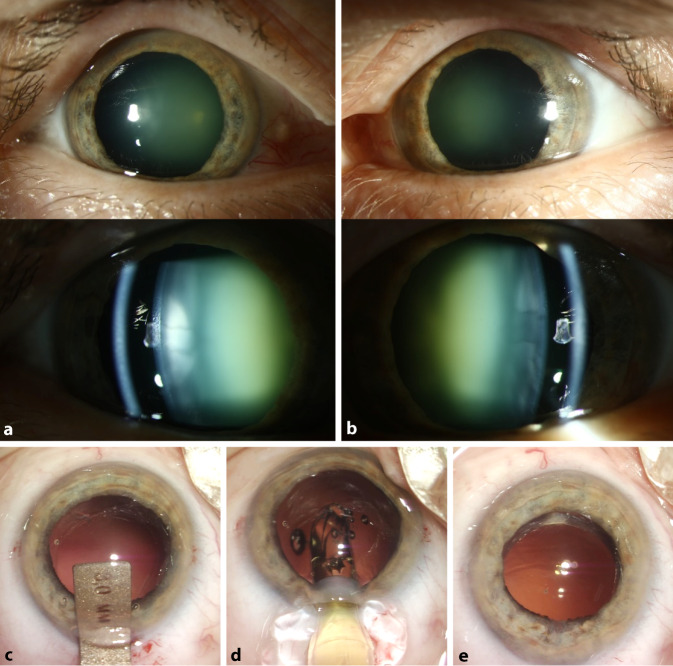

Video 1 zeigt ein Beispiel für eine gelungene Kataraktoperation bei einem 60-jährigen Patienten mit bilateralem Nanophthalmus (Abb. 2). Als zusätzliche Technik wurde ein Femtosekundenlaser für die Kapsulotomie und Fragmentierung der Linse verwendet. Die Hauptinzision wurde temporal platziert. Vor der Operation wurde mithilfe des IOLMaster 700 (Carl Zeiss AG, Oberkochen, Deutschland) die benötigte Brechung der Linse nach mehreren Formeln berechnet (Haigis, Holladay 1, Holladay 2, SRK/T). Die Ergebnisse der Formeln zeigten gravierende Unterschiede, was die Wichtigkeit des Vergleichs mehrerer Formeln bei kleinen Augen aufzeigt (Tab. 2). Eine Linse mit +56 dpt wurde gewählt. Der postoperative Verlauf gestaltete sich problemlos. Dieser kurze Fallbericht soll zeigen, dass die Kataraktchirurgie bei nanophthalmischen Augen bei gründlicher Vorbereitung komplikationsfrei und erfolgreich durchgeführt werden kann. Einen ausführlichen Fallbericht der Operationen präsentiert Naujokaitis et al. [9].

**Table 2:** 

Formel	Rechtes Auge	Linkes Auge
**Berechnete IOL-Power für Emmetropia**		
Hoffer Q	+70,09 dpt	+69,96 dpt
Haigis	+55,28 dpt	+57,47 dpt
SRK/T	+56,04 dpt	+57,09 dpt
Holladay 1	+57,07 dpt	+59,20 dpt
Holladay 2	+57,43 dpt	+59,05 dpt
**Fehler der Vorhersage ***(postoperatives sphärisches Äquivalent – Zielrefraktion)*		
Hoffer Q	−7,57 dpt	−7,75 dpt
Haigis	+1,21 dpt	+0,06 dpt
SRK/T	+0,60 dpt	+0,34 dpt
Holladay 1	−0,19 dpt	−1,29 dpt
Holladay 2	−0,45 dpt	−1,18 dpt

Große Abweichungen zwischen den Formeln sind ersichtlich

Der Vergleich der Formeln erlaubt eine bessere Einschätzung der nötigen Brechkraft der Kunstlinse

## Fazit für die Praxis

- Eine Messung der Achsenlänge sowie der Vorderkammertiefe ist präoperativ nötig, um Patienten mit veränderter Vorderkammermorphologie zu erkennen (Scheimpflug, Vorderabschnitts-OCT).
- Step-by-Step-Chop, Posterior-Plane-Emulsifikation und Soft-Shell-Technik sind Methoden, um die Phakoemulsifikation sicherer zu gestalten und den Endothelzellverlust zu senken.
- Modifizierte Formeln für kurze Achsenlängen sollten zur IOL-Planung verwendet werden und miteinander verglichen werden.
- Die Kataraktoperation in klein gebauten Augen sollte von erfahrenen Chirurgen durchgeführt werden.

## Supplementary Information




